# Supplementary material for: LuTHy: a double‐readout bioluminescence‐based two‐hybrid technology for quantitative mapping of protein–protein interactions in mammalian cells
Source: Mol Syst Biol. 2018 Jul 11;14(7):e8071. doi: 10.15252/msb.20178071 (PMC6039870; doi:10.15252/msb.20178071)
Supplement: Supplementary file 1 — Appendix [file MSB-14-e8071-s001.pdf]

Table of Contents

Appendix Figure S1: Requirements for LuTHy assays.

Appendix Figure S2: LuTHy assay principle.

Appendix Figure S3: Expression values for the interaction PA-mCit-BAD and NL-BCL2L1.

Appendix Figure S4: Quantification of cBRET and cLuC ratios for binary interactions in hPRS.

Appendix Figure S5: Quantification of cBRET and cLuC ratios for binary interactions in hRRS.

Appendix Figure S6: Comparision of the double-readout LuTHy method with single-readout PPI detection methods.

Appendix Figure S7: Quantification of cBRET and cLuC ratios for binary intractions in AIRS.

Appendix Figure S8: Analysis of the interaction between RNASE1 and RNH1 with LuTHy, FRET and DULIP.

Appendix Figure S9: Investigating the binding strengths of interactions from the AIRS with donor saturation BRET assays.

Appendix Figure S10: Analyzing the effects of missense mutations on the interaction between Munc18 and Syntaxin-1 with BRET and BLI.

Appendix Figure S11: Detecting the interaction between NL-VCP and UBXD9-mCit with BRET at near endogenous levels.

Appendix Figure S12: Reproducibility of the targeted CSPα interaction screen.

Appendix Figure S13: Quantification of cBRET and cLuC ratios from the primary screen of CSPα against the synaptic library.

Appendix Figure S14: Investigation of CSPα and PICK1 interactions with LuTHy and proximity ligation assays.

Appendix Figure S15: Effects of disease-causing mutations on the detection of CSPα interactions with LuTHy.

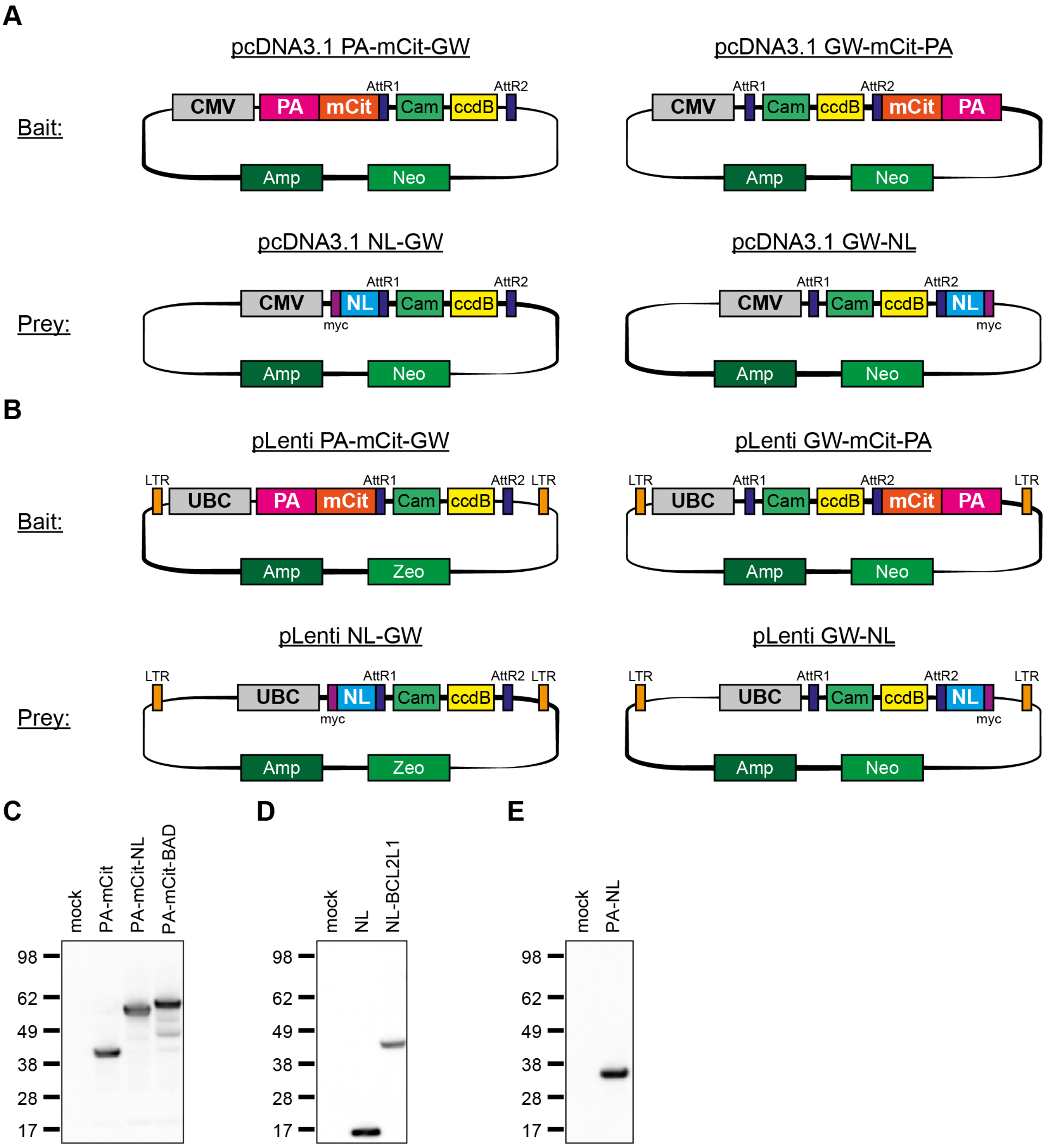

F

$$Cf = \frac{LWL_{PA\text{-}NL}}{SWL_{PA\text{-}NL}}$$

$$\text{BRET ratio} = \frac{LWL}{SWL} - Cf$$

$$\text{cBRET ratio} = \text{BRET ratio}_{PPI} - \uparrow\text{BRET ratio}_{C1/C2}$$

G

$$PIR_{PA\text{-}NL} = \frac{NL_{OUT}}{3 \times NL_{IN}}$$

$$\text{LuC ratio} = \frac{NL_{OUT} / 3 \times NL_{IN}}{PIR_{PA\text{-}NL}}$$

$$\text{cLuC ratio} = LuC_{PPI} - \uparrow LuC_{C1/C2}$$

Appendix Figure S1: Requirements for LuTHy assays.

A,B LuTHy vectors generated for PPI analysis by transient transfection (**A**) or lentiviral transduction (**B**). Gateway compatible vectors for the production of PA-mCit- or NL-tagged fusion proteins. The Gateway cassette (GW) is flanked by AttR1 and AttR2 sites that allow the introduction of AttL1 and AttL2 flanked open-reading frames by LR recombination reactions. Bait (acceptor) vectors produce N- or C-terminally tagged ProteinA (PA)-mCitrine (mCit) fusion proteins. Prey (donor) vectors produce N- or C-terminally tagged NanoLuc (NL) fusion proteins that additionally harbor a myc epitope tag. CMV: cytomegalovirus promoter; UBC: ubiquitin C promoter; Amp: ampicillin resistance; Neo: neomycin resistance; Zeo: zeocin resistance; Cam: chloramphenicol resistance; ccdB: ccdB gene; LTR: long terminal repeat.

C-E Immunoblots of transiently transfected HEK293 cells producing the LuTHy control proteins PA-mCit, PA-mCit-NL, NL, PA-NL and the fusion proteins PA-mCit-BAD and NL-BCL2L1. Immunoblots were detected with an α-GFP (**C**) or an α-NL (**D,E**) antibody.

F Calculation of BRET and cBRET ratios for PPI detection experiments. The correction factor (Cf) for the donor bleed-through is calculated from the luminescence emission of the PA-NL construct at the LWL divided by the SWL. BRET ratios are calculated from the luminescence emission at the LWL divided by the SWL followed by subtraction of the Cf. Finally, the corrected BRET (cBRET) ratio is calculated by subtracting the higher BRET ratio of the two control interactions (NL-X/PA-mCit and NL/PA-mCit-Y) from the BRET ratio of the interaction of interest (NL-X/PA-mCit-Y).

G Calculation of the LuC and cLuC ratios for PPI detection. The precipitation ratio ($PIR_{PA\text{-}NL}$) is calculated from the total luminescence emission of the PA-NL construct after co-precipitation (NL_{OUT}) divided by the total luminescence emission in cell lysates (NL_{IN}). LuC ratios for all tested interactions are calculated from the total luminescence emission after co-precipitation (NL_{OUT}) divided by the total luminescence emission in cell lysates (NL_{IN}) normalized to the $PIR_{PA\text{-}NL}$. Finally, the corrected LuC (cLuC) ratios are calculated by subtracting the higher LuC ratio of the two control interactions (NL-X/PA-mCit and NL/PA-mCit-Y) from the LuC ratio of the interaction of interest (NL-X/PA-mCit-Y).

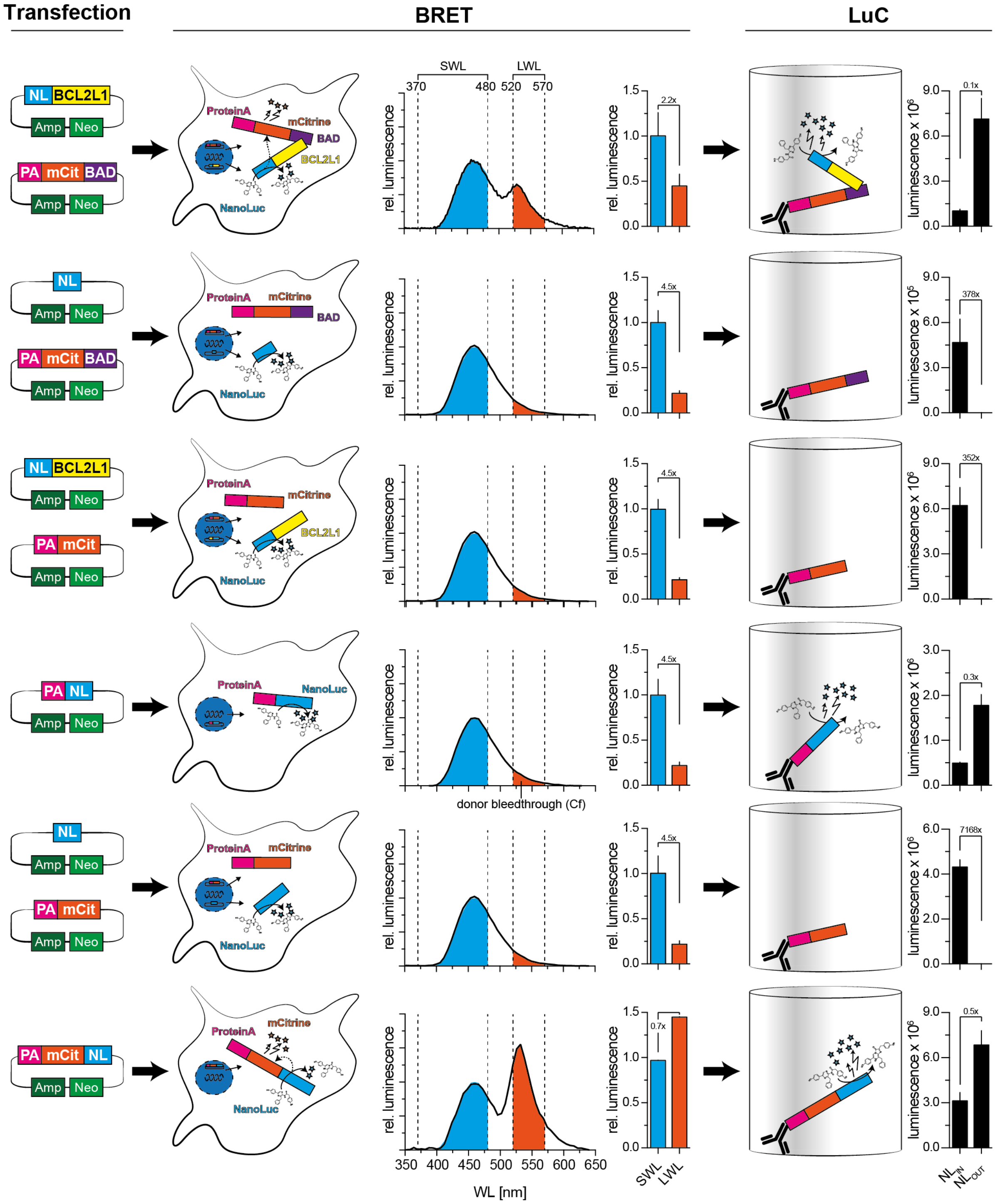

Appendix Figure S2: LuTHy assay principle.

The interaction between PA-mCit-BAD and NL-BCL2L1 is used as example. Initially, the indicated constructs are transfected into mammalian cells. Luminescence scan measurements illustrate the energy transfer from the donor (NanoLuc luciferase) to the fluorescent acceptor (mCitrine). Luminescence scan measurements from 350-650 nm display two emission peaks at ~460 and ~530 nm for the interaction PA-mCit-BAD/NL-BCL2L1 but only one at 460 nm for the controls PA-mCit-BAD/NL and PA-mCit/NL-BCL2L1. For the PA-NL fusion construct and the negative interaction between PA-mCit/NL one emission peak at ~460 nm is visible. In contrast, the tandem construct PA-mCit-NL displays two emission peaks at ~460 and ~530 nm. The luminescence emission at the short wavelength (SWL, 370-480 nm) and the long wavelength (LWL, 520-570 nm) of the PA-NL control construct is used to determine the correction factor for the donor bleed-through necessary to calculate the BRET ratio for a studied interaction. Finally, cells are lysed and the luminescence-based co-precipitation (LuC) is performed. To calculate LuC ratios, NanoLuc emission after co-precipitation (NL_{OUT}) is set in relation to its emission in cell lysates (NL_{IN}).

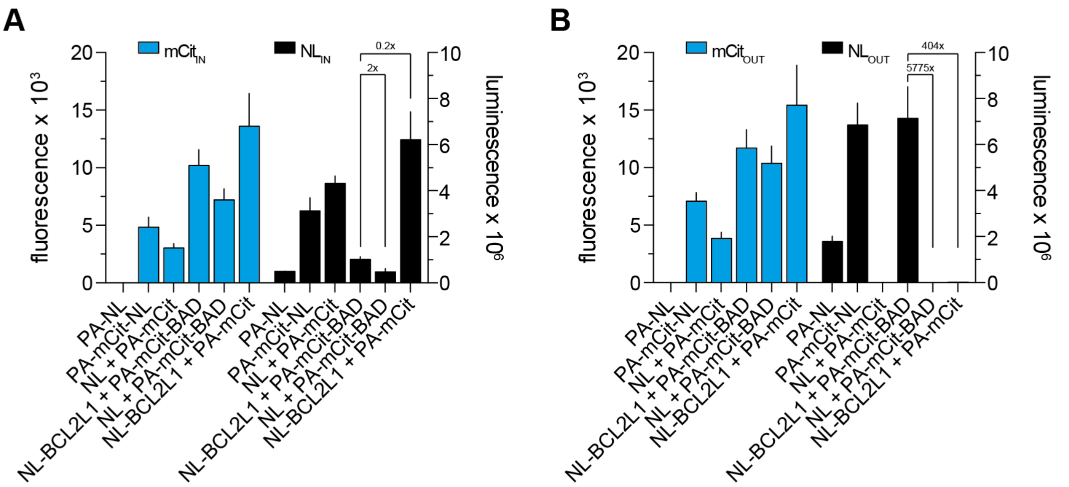

Appendix Figure S3: Expression values for the interaction PA-mCit-BAD and NL-BCL2L1.

A Protein expression of all constructs is detected by measuring the fluorescence emission of mCit (mCit$_{IN}$) and the total luminescence emission of NL (NL$_{IN}$) after cell lysis.

B Precipitation of PA-tagged proteins is confirmed by measuring the fluorescence emission of mCit (mCit$_{OUT}$); detection of NL luciferase activity (NL$_{OUT}$) indicates protein complex formation. Data are representative of more than three independent experiments. All values are mean ± sd.

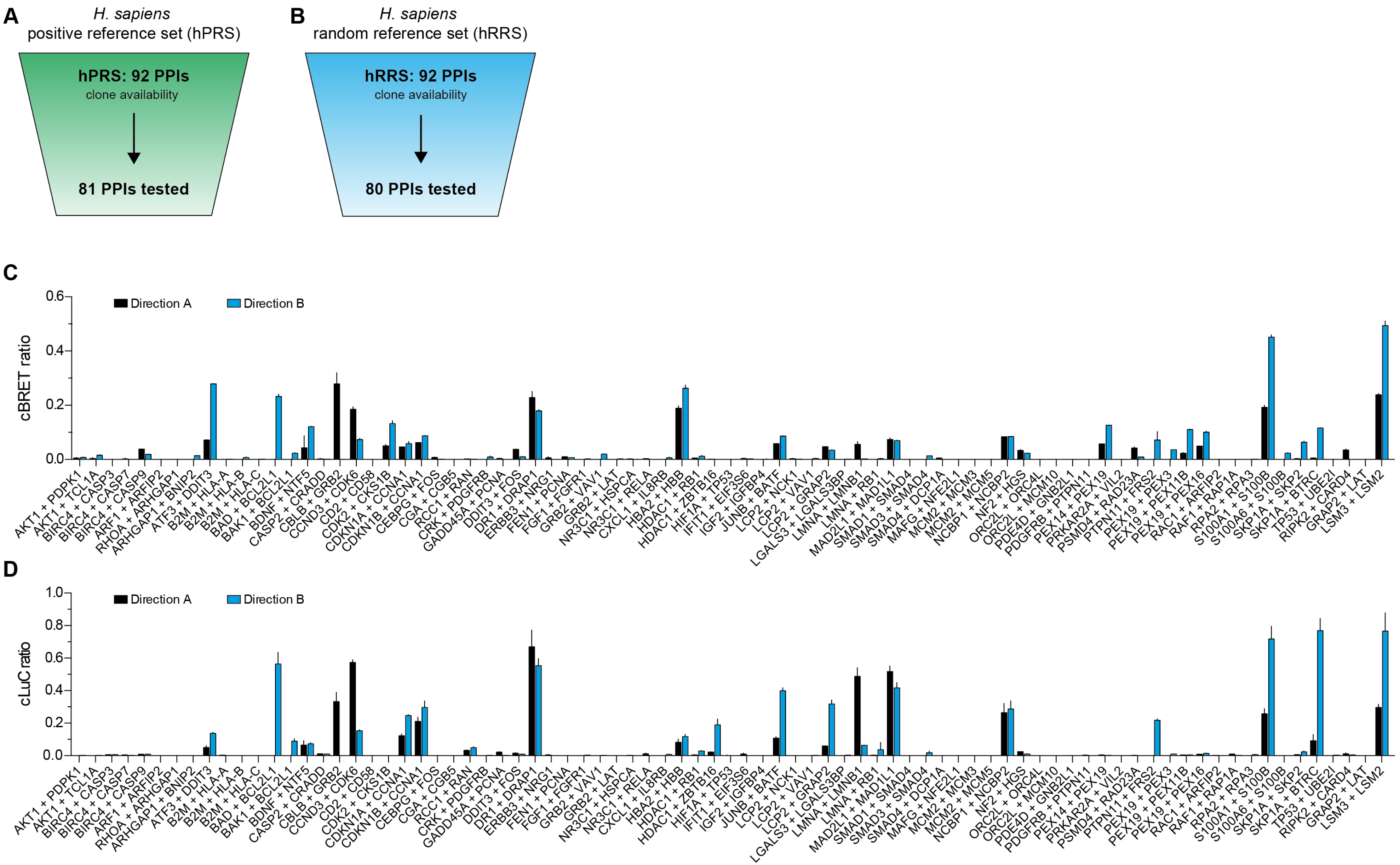

Appendix Figure S4: Quantification of cBRET and cLuC ratios for binary interactions in hPRS.

A,B Selection strategy for 92 interactions from the H. sapiens positive reference set (hPRS) and H. sapiens random reference set (hRRS). Due to cDNA clone availability and LR recombination success 81 PPIs of the hPRS (**A**) and 80 PPIs of the hRRS (**A**) were tested with the LuTHy assay.

C,D cBRET (**C**) and cLuC (**D**) ratios for the PPIs in the reference set were determined for direction A (bait/prey: NL-X/PA-mCit-Y) and direction B (prey/bait: NL-Y/PA-mCit-X). All values are mean ± sem from two independent experiments.

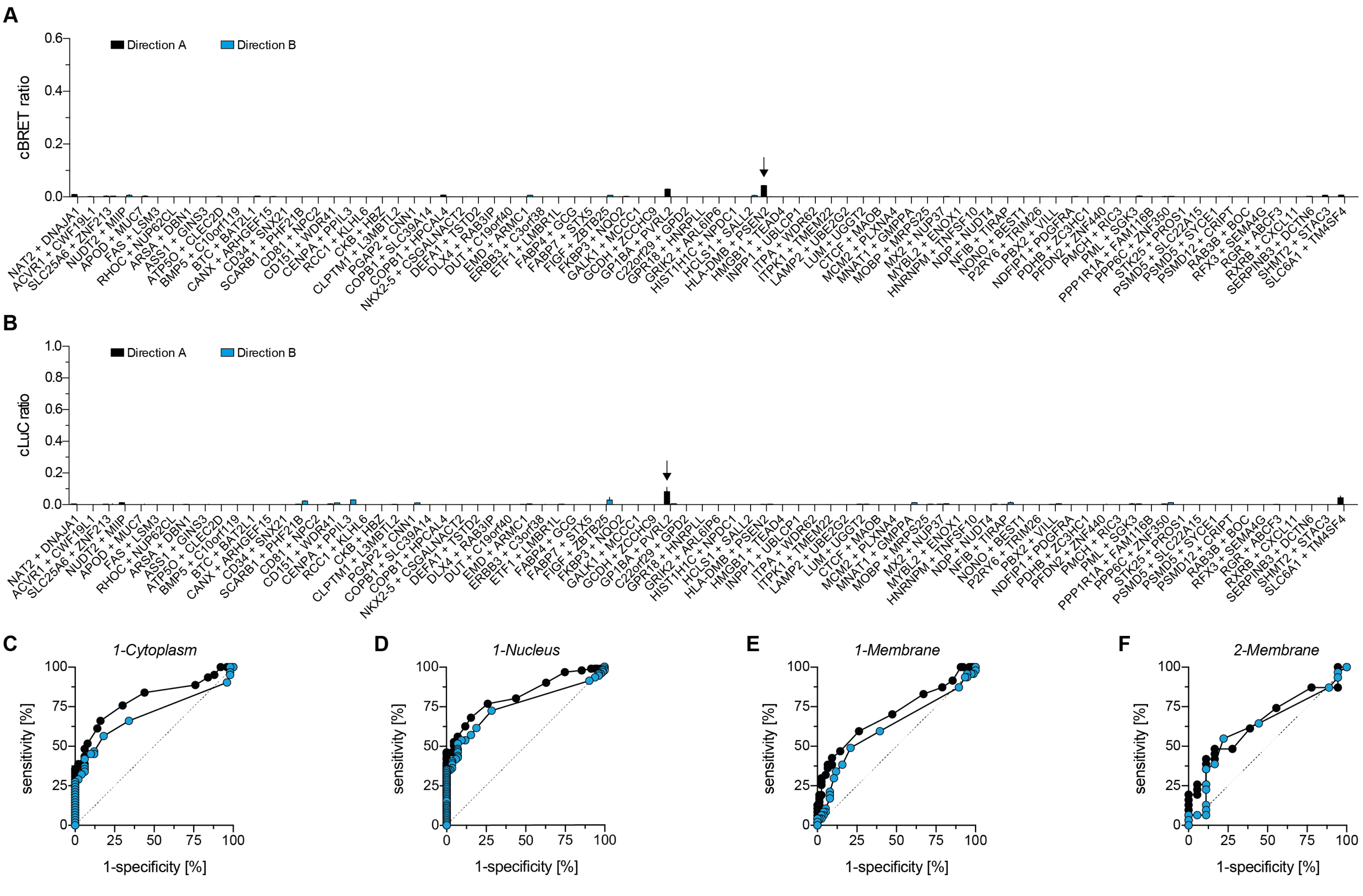

Appendix Figure S5: Quantification of cBRET and cLuC ratios for binary interactions in hRRS.

A,B cBRET (**A**) and cLuC ratios (**B**) for the PPIs in hRRS were determined for direction A (bait/prey: NL-X/PA-mCit-Y) and direction B (prey/bait: NL-Y/PA-mCit-X). All values are mean ± sem from two independent experiments.

C-F ROC analysis of cBRET and cLuC data from the hPRS and hRRS for interactions with at least one protein located in the cytoplasm (1-Cytoplasm), nucleus (1-Nucleus), membrane (1-Membrane) or both proteins located at the membrane (2-Membrane).

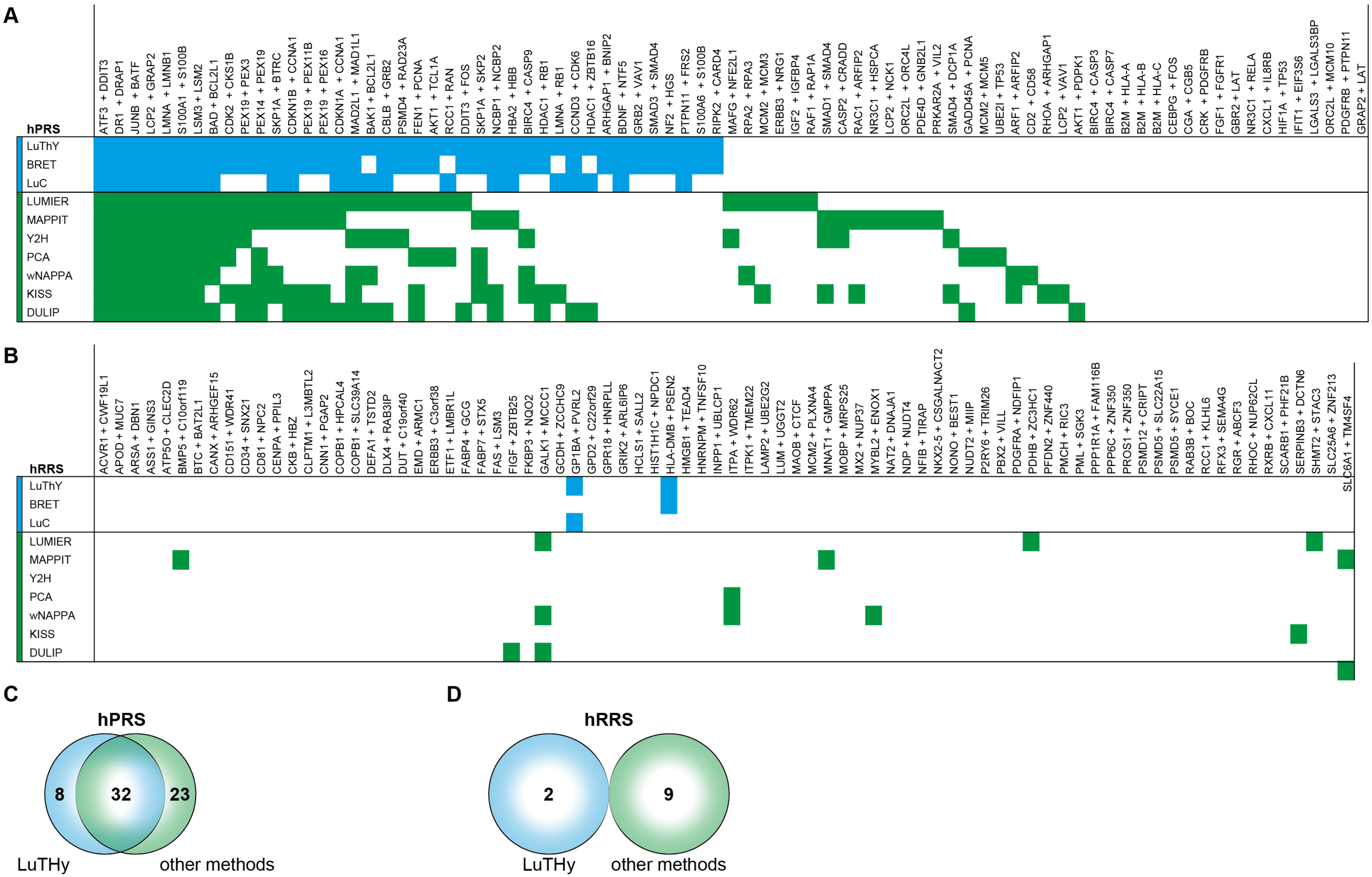

Appendix Figure S6: Comparison of the double-readout LuTHy method with single-readout PPI detection methods.

A,B Interactions from the hPRS (**A**) and hRRS (**B**) detected with the LuTHy method in comparison to previously published methods (Braun et al, 2009; Lievens et al, 2014; Trepte et al, 2015).
C,D Venn diagrams depicting the overlap between the number of interactions detected in the hPRS (**C**) and hRRS (**D**) with LuTHy and previously published methods (Braun et al, 2009; Lievens et al, 2014; Trepte et al, 2015).

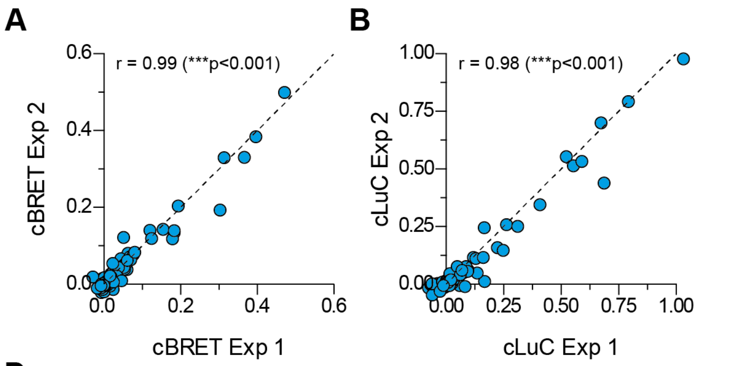

C

K_D [M]	AIRS	LuThY pos	BRET pos	LuC pos	Double pos
10^{0} - 10^{-6}	28	14	12	5	3
10^{-6} - 10^{-8}	27	12	12	6	6
10^{-8} - 10^{-16}	16	9	7	8	6
**Total**	**71**	**35**	**31**	**19**	**15**

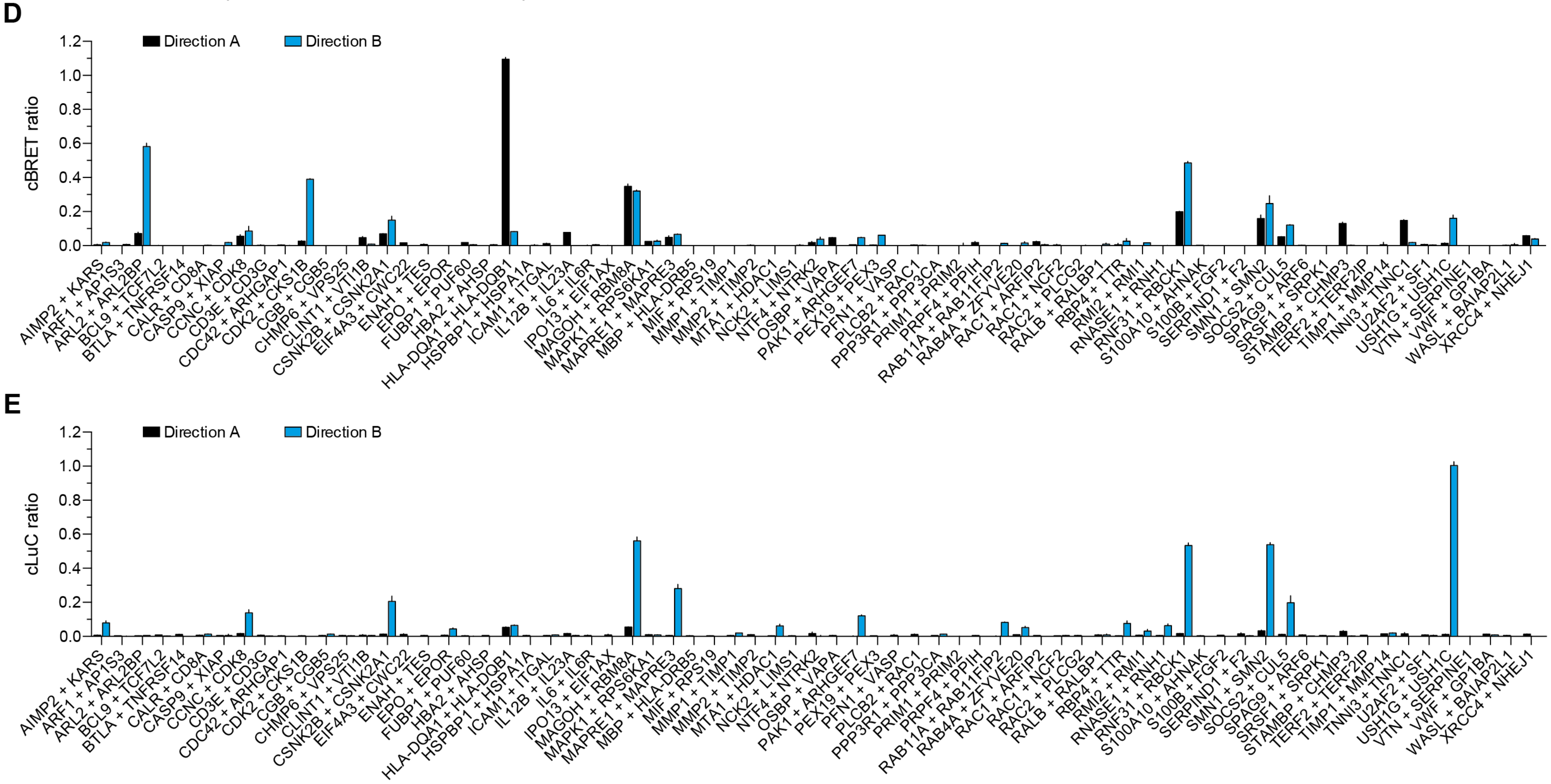

Appendix Figure S7: Quantification of cBRET and cLuC ratios for binary interactions in AIRS.

A,B Reproducibility of cBRET and cLuC measurements; binary interactions from AIRS were systematically analyzed. The scatter plots show the mean cBRET (**A**) or cLuC (**B**) ratios from two independent experiments (Exp 1 and Exp 2); the calculated two-tailed Pearson correlation coefficients are indicated; ***p<0.001.

C Number of tested PPIs, their respective binding affinity range and numbers of interactions detected with BRET and LuC assays.

D,E cBRET (**D**) and cLuC (**E**) ratios for PPIs in the AIRS determined for direction A (bait/prey: NL-X/PA-mCit-Y) and direction B (prey/bait: NL-Y/PA-mCit-X). All values are mean ± sem from two independent experiments.

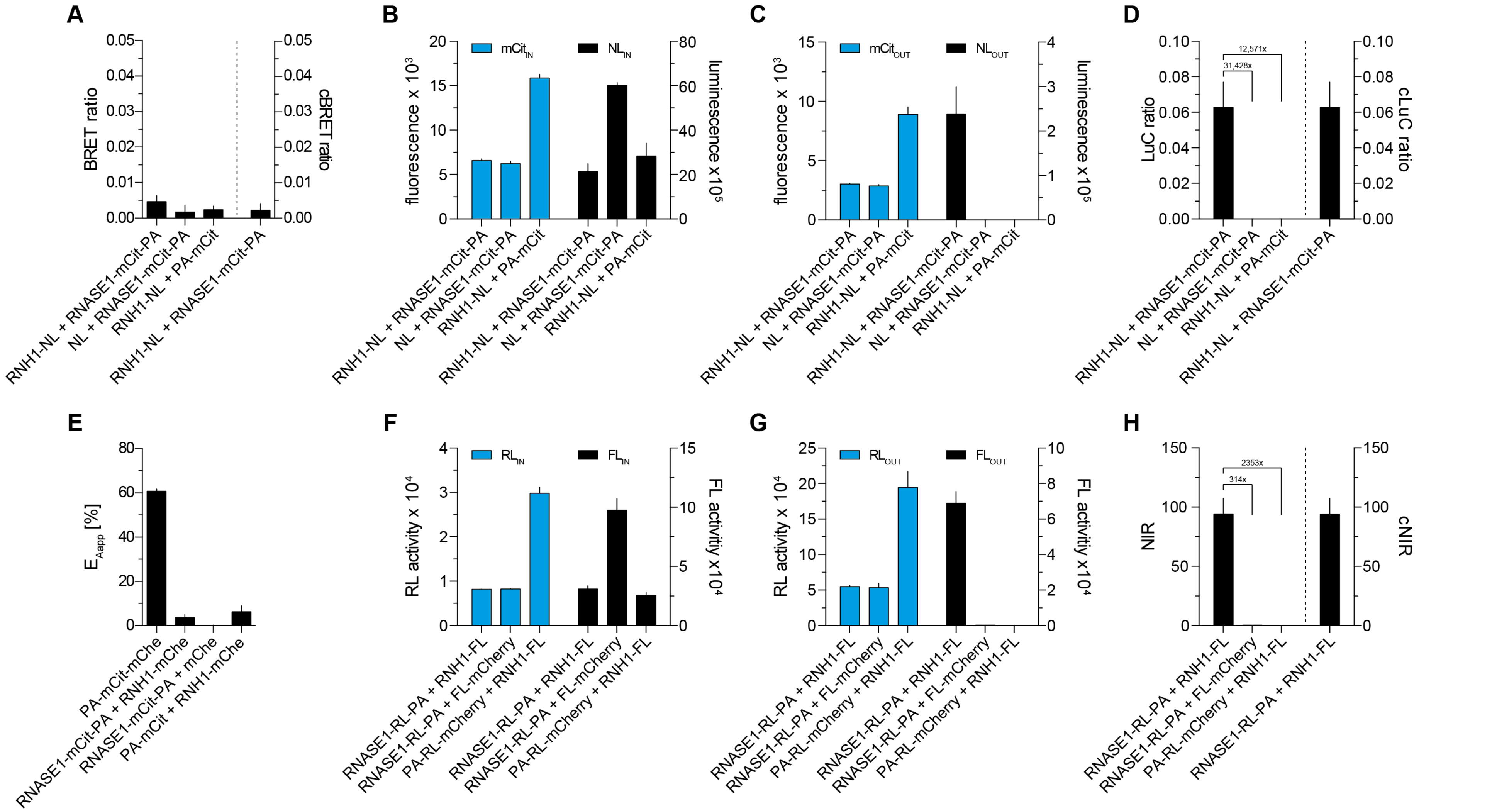

Appendix Figure S8: Analysis of the interaction between RNASE1 and RNH1 with LuTHy, FRET and DULIP.

A BRET and cBRET ratios for the indicated interactions.
B Fluorescence (mCit$_{IN}$) and luminescence (NL$_{IN}$) values measured in cell lysates.
C Fluorescence (mCit$_{OUT}$) and luminescence (NL$_{OUT}$) values measured after co-precipitation.
D Calculated LuC and cLuC values for the indicated interactions.
E FRET efficiencies (E_{Aapp}) obtained for the tested interactions; the mCitrine-mCherry fusion protein was used as a positive control.
F Renilla (RL$_{IN}$) and firefly (FL$_{IN}$) luciferase activities measured in cell lysates with the DULIP assay.
G Renilla (RL$_{OUT}$) and firefly (FL$_{OUT}$) luciferase activities measured after co-precipitation with DULIP.
H NIR and cNIR values were calculated for the tested interactions. All values are mean ± sem from two independent experiments.

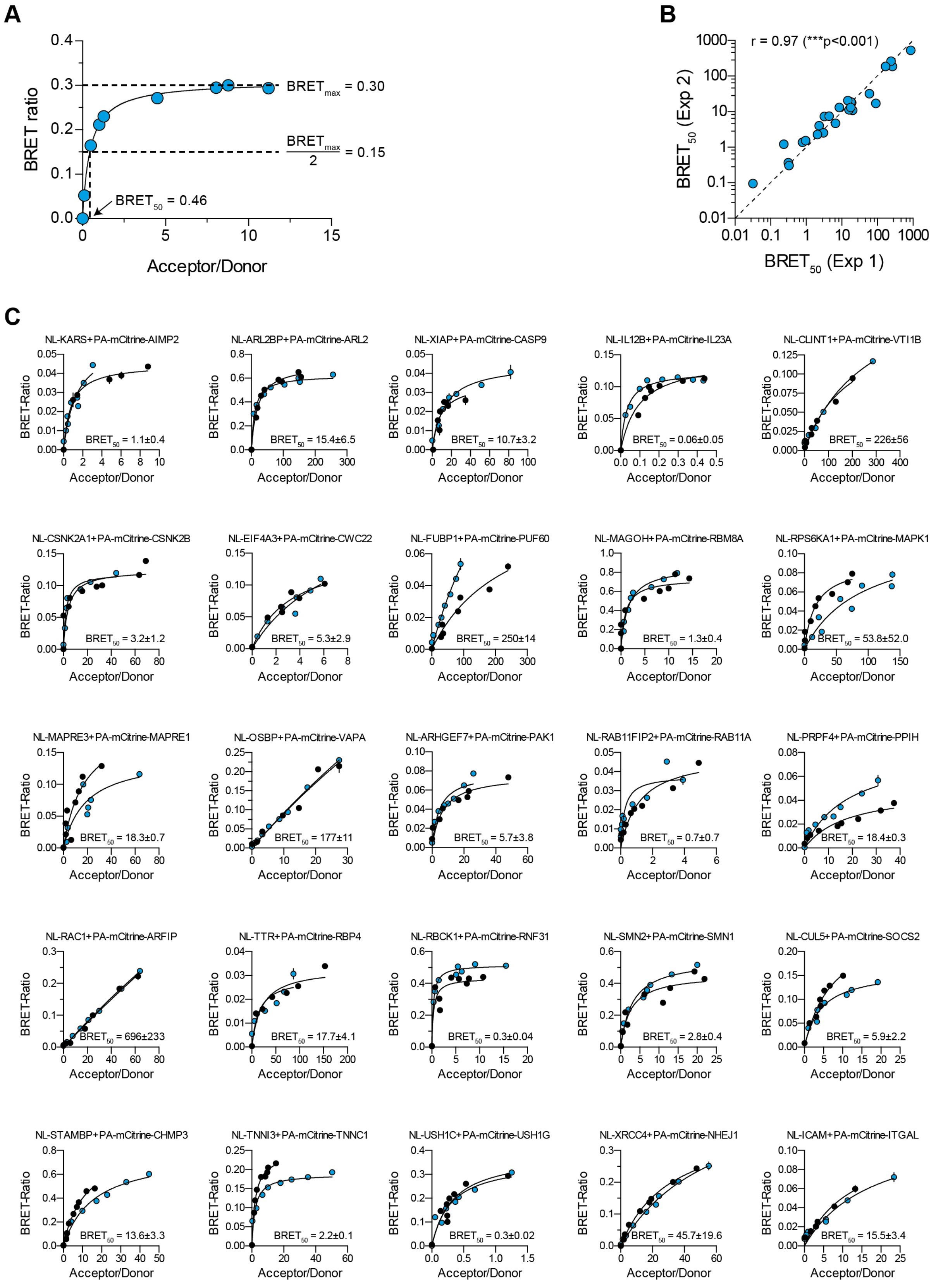

Appendix Figure S9: Investigating the binding strengths of interactions from the AIRS with donor saturation BRET assays.

A Donor saturation BRET experiment for the interaction between PA-mCit-BAD and NL-BCL2L1. Increasing concentrations of the plasmid encoding PA-mCit-BAD were co-transfected with a constant amount of the plasmid encoding NL-BCL2L1 in HEK293 cells. Non-linear curve fitting was applied to determine BRETmax and BRET50 values.

B Donor saturation assays; 25 interactions in AIRS were assessed with the BRET readout. BRET50 values were obtained with high reproducibility in two independent experiments. Significance was calculated by two-tailed Pearson correlation. ***p<0.001.

C Results from donor saturation assays. 25 interactions selected from AIRS were systematically assessed. For all PPIs two independent experiments were performed. BRET50 values were determined by non-linear curve fitting and the mean ± sd indicated.

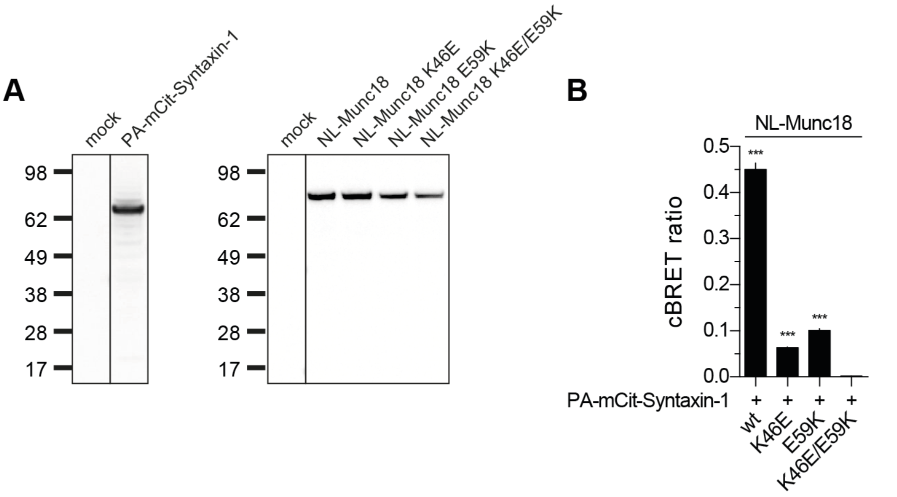

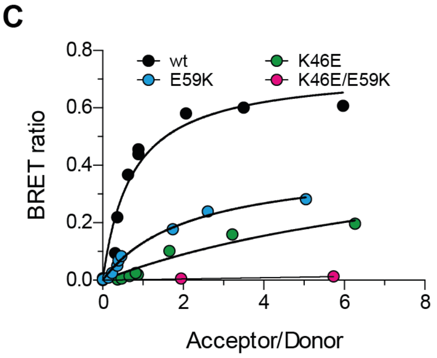

D

Interaction	K_D (nM)[1]	$BRET_{50}$
Syntaxin-1 + Munc18 wt	23.3±11.1	0.73±0.10
Syntaxin-1 + Munc18 K46E	181.2±46.4	10.11±4.16
Syntaxin-1 + Munc18 E59K	83.9±34.3	2.17±0.21
Syntaxin-1 + Munc18 K46E/E59K	-	-

[1]Han et al., 2011

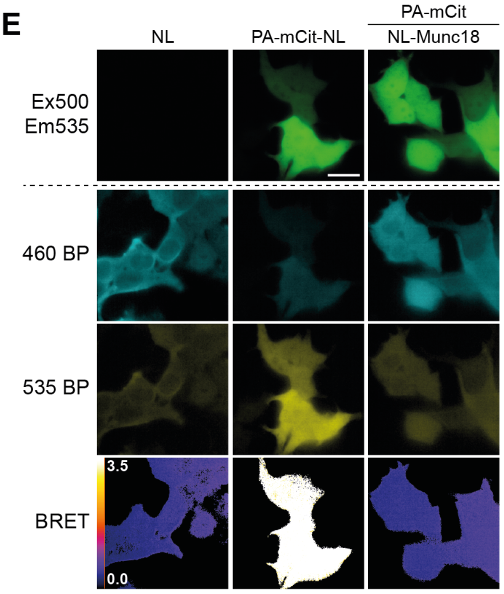

Appendix Figure S10: Analyzing the effects of missense mutations on the interaction between Munc18 and Syntaxin-1 with BRET and BLI.

A Immunoblots of HEK293 cells producing the fusion proteins PA-mCit-Syntaxin-1 (anti-GFP) or the fusion proteins NL-Munc18-wt, -K46E, -E59K and -K46E/E59K (anti-NanoLuc).

B Effects of single missense mutations (K46E and E59K) and a double mutation (K46E/E59K) in Munc18 on its association with Syntaxin-1. Binary interactions were monitored with the BRET readout. Data are representative of more than three independent experiments. All values are mean ± sd. Significance was calculated by one-way ANOVA followed by Dunnett's multiple comparisons post-hoc test; ***p<0.001.

C Donor saturation assays to determine the effect of missense mutations on BRET50. Constant amounts of NL-Munc18 variants (wt, K46E, E59K, K46E/E59K) were co-expressed with increasing amounts of PA-mCit-Syntaxin-1 in HEK293 cells. Non-linear curve fitting was performed to obtain BRET50 values. Data are representative of more than three independent experiments. All values are mean ± sd.

D Table of published binding affinities for the indicated interactions between Syntaxin-1 and wt Munc18 as well as its mutant variants Munc18-K46E, -E59K and -K46E/E59K (Han et al, 2011). The BRET50 values determined in this study with donor saturation BRET assays are also depicted.

E Live-cell bioluminescence imaging (BLI) of HEK293 cells co-producing the indicated fusion proteins. mCitrine was excited at 500 nm, emitted fluorescence was detected at 535 nm. After substrate addition, short (460) and long (535) band-pass (BP) filters in a dual-view adapter were used to detect the emitted luminescence simultaneously at the respective wavelengths. BRET images were calculated by dividing the 535 BP by the 460 BP images using ImageJ. Scale bar = 20 µm.

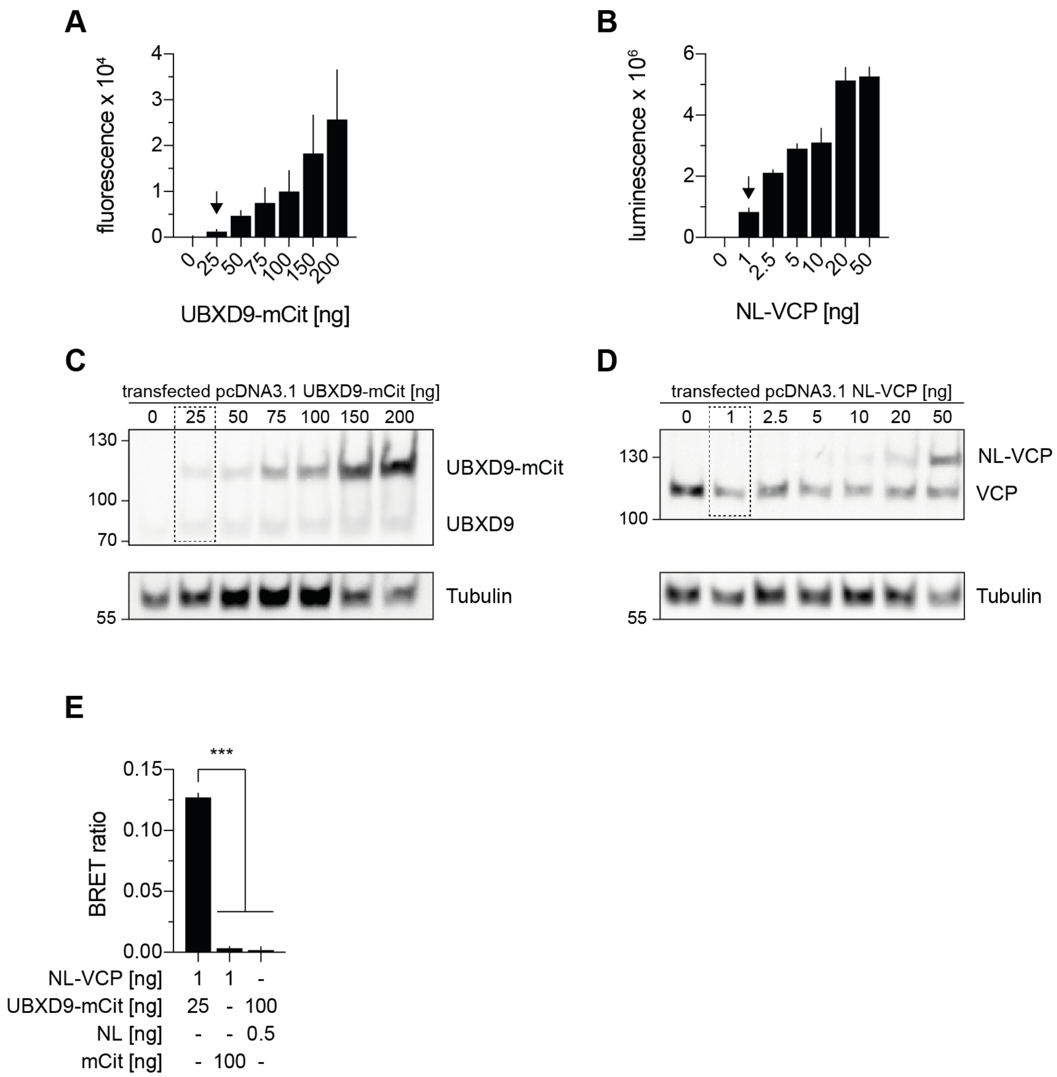

Appendix Figure S11: Detecting the interaction between NL-VCP and UBXD9-mCit with BRET at near endogenous protein levels

A-D Increasing amounts of DNA encoding UBXD9-mCit (**A,C**) or NL-VCP (**B,D**) were transfected into HEK293 cells, respectively, and the fluorescence (**A**) or bioluminescence (**B**) detected. After 48h cells were lysed and analyzed by SDS PAGE and immunoblotting. Immunoblots show endogenous UBXD9 (**C**) and VCP (**D**) levels in comparison to protein levels of recombinantly expressed proteins UBXD9-mCit (**C**) and NL-VCP (**D**).

E BRET measurements of cells co-transfected with 25 ng of pcDNA3.1-UBXD9-mCit and 1 ng pcDNA3.1-NL-VCP. As controls, cells co-transfected pcDNA3.1-NL-VCP and pcDNA3.1-mCit or with pcDNA3.1-UBXD9-mCit and pcDNA3.1-NL were analyzed. All values are mean ± sd.

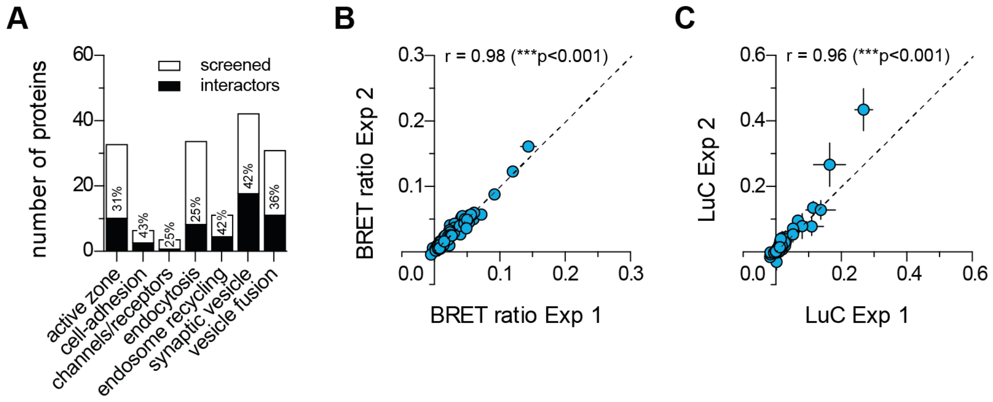

Appendix Figure S12: Reproducibility of the targeted CSPα interaction screen.

A Numbers of screened presynaptic proteins manually annotated according to their function and localization in the presynaptic terminal. The numbers of proteins that were identified with LuTHy to interact with CSP α-mCit-PA are indicated.

B,C Assessment of the reproducibility of PPI detection. BRET (**B**) and LuC (**C**) measurements from two independent PPI detection screens are shown. In each experiment CSPα-mCit-PA was screened against a focused library of 125 NL-tagged presynaptic proteins. The calculated two-tailed Pearson correlation coefficient is indicated; ***p<0.001.

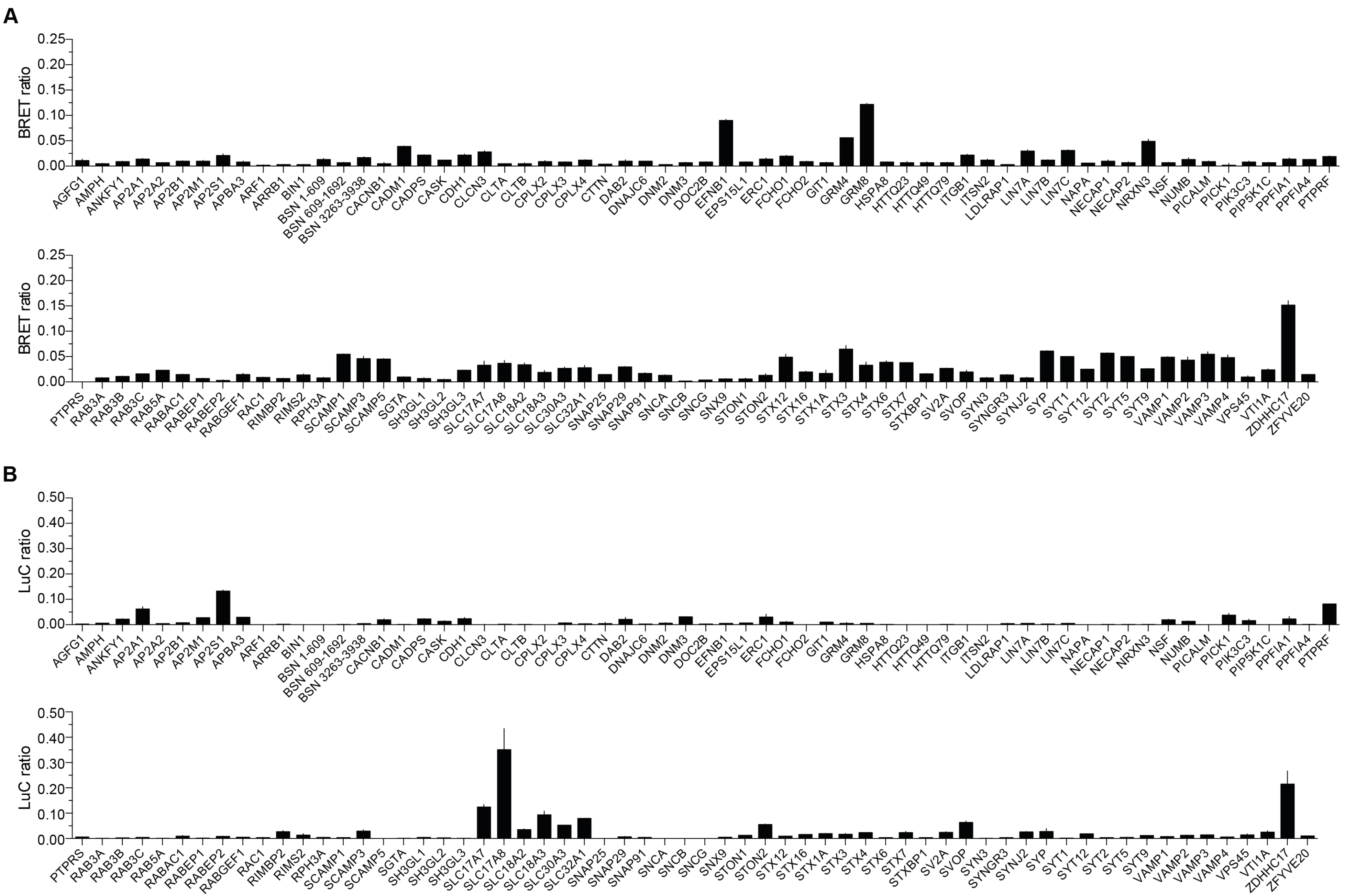

Appendix Figure S13: Quantification of BRET and LuC ratios from the primary screen of CSPα against the synaptic library.

A,B BRET (**A**) and LuC ratios (**B**) for 125 tested binary interactions are shown. All NL-tagged proteins were systematically screened with LuThY for interaction with CSPα-mCit-PA. All values are mean ± sem from two independent experiments.

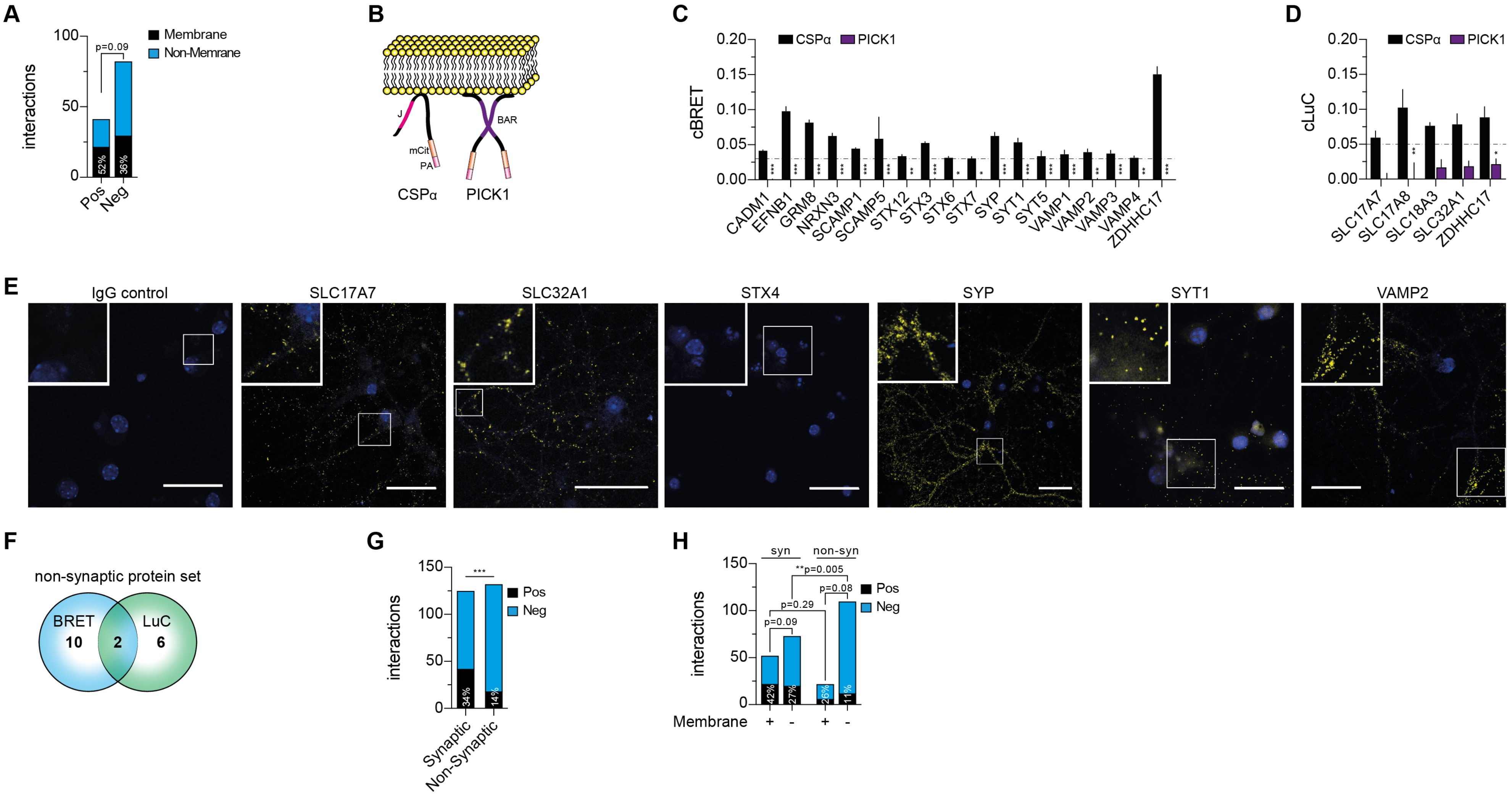

Appendix Figure S14: Investigation of CSPα and PICK1 interactions with LuTHy and proximity ligation assays

A Fraction of interactions with membrane and non-membrane proteins identified as CSPα interaction partners in the synaptic protein set. Significance was calculated by two-sided Fisher's exact test.

B Schematic of the association of CSPα and PICK1 to lipid bilayers by palmitoylation.

C cBRET ratios obtained with systematically tested membrane proteins and CSPα-mCit-PA or PA-mCit-PICK1. All values are mean ± sem from two independent experiments. Significance was calculated by two-way ANOVA followed by Dunnett's multiple comparisons post-hoc test; *p<0.05, **p<0.005, ***p<0.001.

D cLuC ratios obtained with systematically tested membrane proteins and CSPα-mCit-PA or PA-mCit-PICK1. All values are mean ± sem from two independent experiments. Significance was calculated by two-way ANOVA followed by Dunnett's multiple comparisons post-hoc test; *p<0.05, **p<0.005.

E Representative images obtained from proximity ligation experiments. Primary neurons from mice were co-incubated with primary antibodies against CSPα and either an IgG control antibody or antibodies against SLC17A7, SLC32A1, STX4, SYP, SYT1 or VAMP2. Afterwards PLA probes were added, ligated, amplified and detected with a Cy3 probe. Strong PLA signals were only detected for interactions identified with LuTHy, while no PLA signals were obtained for the IgG control antibody and STX4. Scale bar = 40 µm.

F Venn diagram depicting the 18 identified CSPα interaction partners in the non-synaptic protein set by LuTHy.

G Positive and negative interactions detected in the synaptic and non-synaptic PPI sets. Significance was calculated by two-sided fisher's exact test; ***p<0.001.

H Number of positive and negative interactions of CSPα with membrane and non-membrane proteins in the synaptic and non-synaptic binary interaction sets. CSPα does not interact with significantly more membrane proteins than with non-membrane proteins in the synaptic (p = 0.09) or the non-synaptic protein set (p = 0.08). A significant enrichment for interactions with synaptic over non-synaptic proteins was observed for non-membrane proteins (p = 0.005), but not for membrane proteins (p = 0.29). Significance was calculated by two-sided Fisher's exact test; **p<0.005.

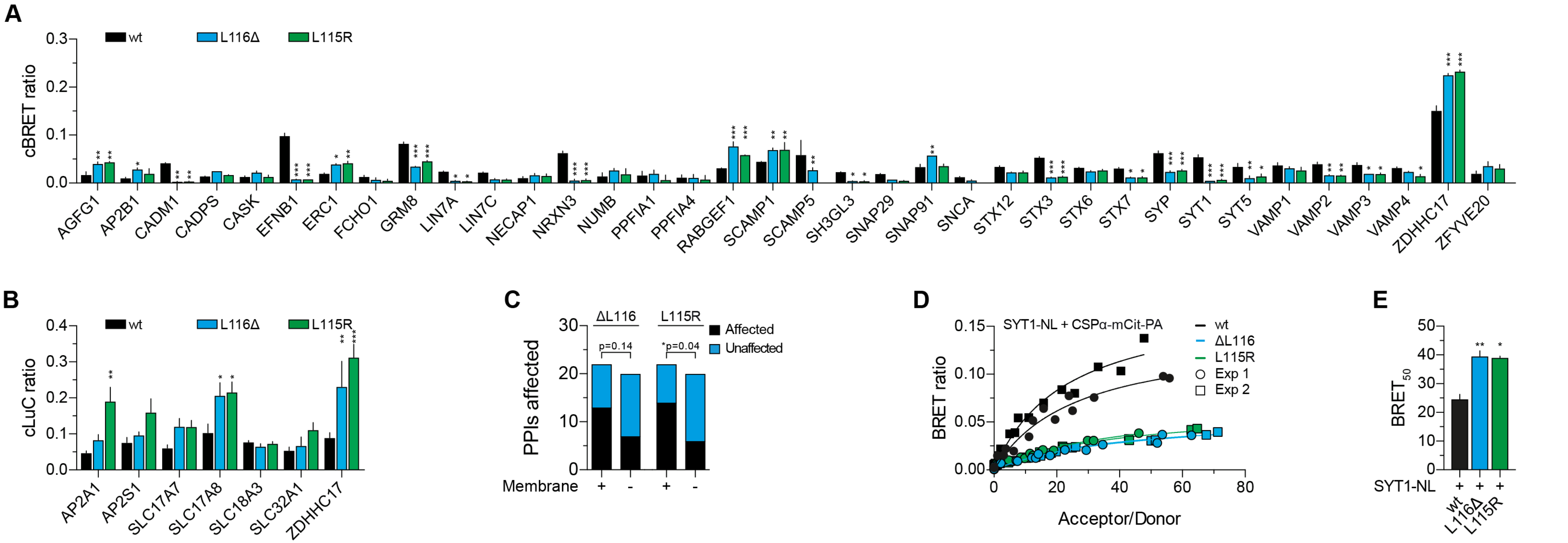

Appendix Figure S15: Effects of disease-causing mutations on the detection of CSPα interactions with LuTHy.

A Detection of binary interactions between wild-type CSPα-mCit-PA or its mutant variants ΔL116 and L115R with NL-tagged presynaptic fusion proteins using BRET assays. All values are mean ± sem from two independent experiments. Significance was calculated by two-way ANOVA followed by Dunnett's multiple comparisons post-hoc test; *p<0.05, **p<0.005, ***p<0.001.

B Detection of binary interactions between wild-type CSPα-mCit-PA or its mutant variants ΔL116 and L115R with NL-tagged presynaptic fusion proteins with LuC assays. All values are mean ± sem from two independent experiments. Significance was calculated by two-way ANOVA followed by Dunnett's multiple comparisons post-hoc test; *p<0.05, **p<0.005, ***p<0.001.

C Number of CSPα-mCit-PA interactions affected by ΔL116 and L115R mutations. Fraction of affected interactions between membrane and non-membrane proteins are depicted. Significance was calculated by two-sided Fisher's exact test; *p<0.05.

D Donor saturation assays for the interactions between SYT1-NL and the CSPα-mCit-PA variants (wt, ΔL116, L115R) performed in two independent experiments to determine the impact of missense mutations on BRET50 values. Non-linear curve fitting was performed to obtain BRET50 values.

E BRET50 values calculated from two independent donor saturation experiments. The interaction between wild-type (wt) CSPα-mCit-PA and its mutant variants (ΔL116 and L115R) and SYT1-NL was analyzed. Significance was calculated by one-way ANOVA followed by Dunnett's multiple comparison post-hoc test; *p<0.05, **p<0.005.
